# Supplementary material for: Hexadecapolar colloids
Source: Nat Commun. 2016 Feb 11;7:10659. doi: 10.1038/ncomms10659 (PMC4753254; doi:10.1038/ncomms10659)
Supplement: Supplementary Information — Supplementary Figures 1-6, Supplementary Note 1-2 and Supplementary References [file ncomms10659-s1.pdf]

Supplementary Figures

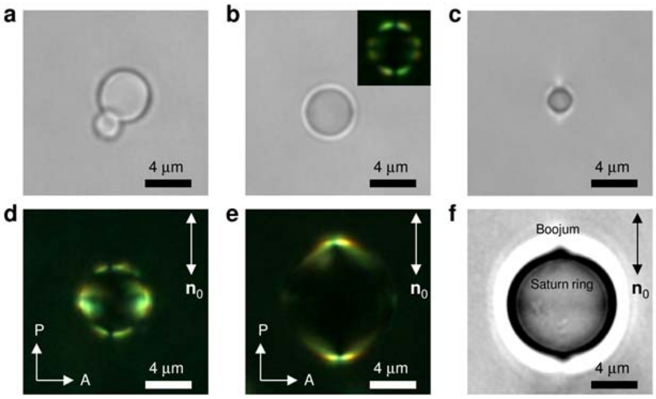

Supplementary Figure 1 | Preparation of colloidal PSMs with conically degenerate anchoring. (**a**-**c**) Optical bright field micrographs showing two initially connected spherical lobes of (**a**) an asymmetric colloidal dimer, which yield (**b**, **c**) two spherical particles after breaking apart, including the larger one (**b**), which spontaneously exhibits conically degenerate surface anchoring; particles in **a**-**c** are imaged on a glass substrate. Inset of **b** shows a POM micrograph of an elastic hexadecapole induced by the larger particle when dispersed in 5CB. (**d**, **e**) Polarizing and (**f**) bright field optical micrographs of the polystyrene divinylbenzene particles of different diameter with conic anchoring obtained after the thermal treatment described in Methods.

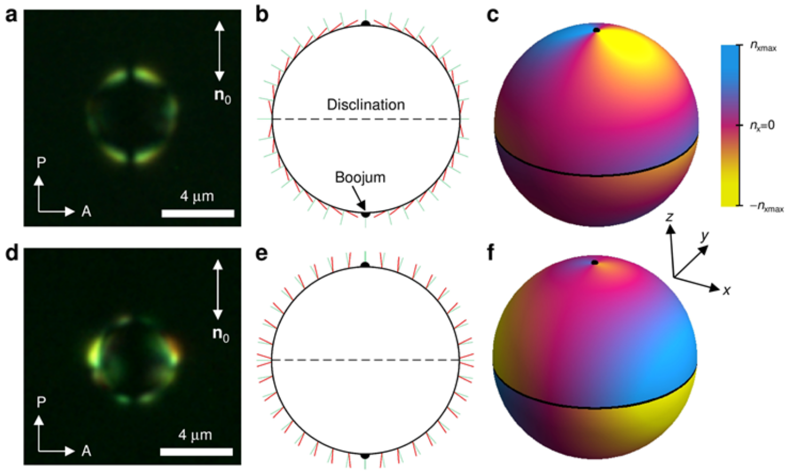

Supplementary Figure 2 | Director field around PSMs with conically degenerate surface anchoring. (**a**, **d**) POM micrographs of elastic hexadecapoles formed around spheres with conic anchoring of a different tilt. (**b**, **e**) Schematic diagrams of the local easy axis for the director (red rods) orientation at the surface of the sphere in the case of (**b**) a relatively large and (**e**) relatively small tilt away from the local surface normal (green rods). (**c**, **f**) Color-coded maps of the director distortions showing n_x at the surface of particles shown, respectively, in **a**, **b** and **d**, **e**.

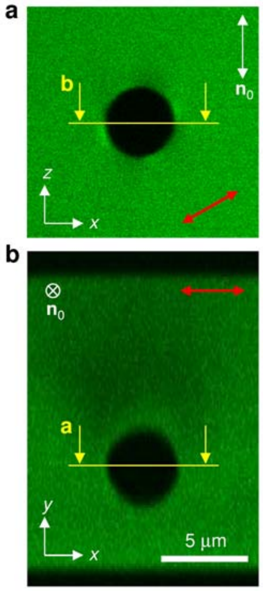

Supplementary Figure 3 | 3PEF-PM images of an elastic hexadecapole in 5CB. The images were obtained in the planes marked on the corresponding cross-sections in **a** and **b**. Red double arrow shows a direction of polarization of the excitation beam.

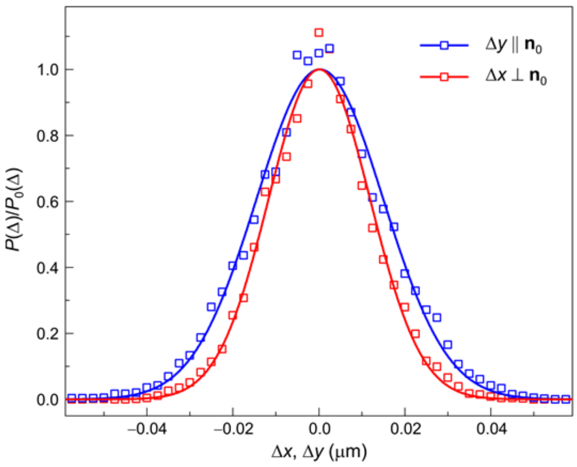

Supplementary Figure 4 | Normalized histograms of hexadecapole's displacements. Displacements along (Δy) and perpendicular (Δx) to the far-field director $\mathbf{n}_0$ were characterized at the frame rate of 15 fps. The open square symbols show experimental data and the solid lines are the corresponding Gaussian fits.

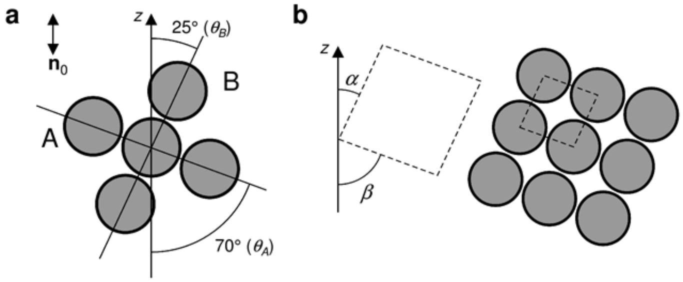

Supplementary Figure 5 | Two-dimensional assembly of hexadecapolar colloids. (**a**) Because of the "steric" effects combined with anisotropic interactions, every particle can have no more than four neighbors. (**b**) Planar colloidal lattice with a rhombic elementary cell shown by the dashed lines. Its free energy depends on the orientation which can be unambiguously defined by the angles α and β, with the anisotropic interactions expected to yield minima at $\alpha \approx \theta_1$ and $\beta \approx \theta_2$.

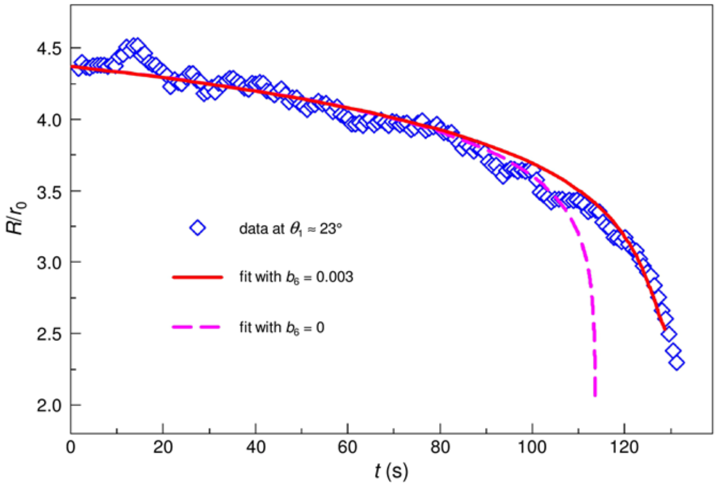

Supplementary Figure 6 | Role of the higher order terms in fitting the separation versus time data. Center-to-center particle separation R along the direction at $\theta_1 \approx 23°$ (average angle on contact) with respect to n_0. The dashed magenta line shows a least-squares fit with $R(t)$ obtained from the simplified equation of motion with only two adjustable parameters $(b_2, b_4) = (-0.026, -0.071)$. The solid red line corresponds to the solution with three adjustable parameters $(b_2, b_4, b_6) = (-0.017, -0.092, 0.003)$.

Supplementary Notes

Supplementary Note 1 - Analysis of colloidal lattices

To explore the structural possibilities for colloidal self-organization of elastic hexadecapoles, let us first consider a two-dimensional system. The pairwise interactions between the hexadecapolar NLC colloids are highly anisotropic. At a fixed inter-particle distance, the interaction energy U_{int} as a function of the angle θ has eight minima (Fig. 2). However, since the particles are not pointwise objects and exclude volume around them that becomes inaccessible to other particles, only four of these minima can be occupied simultaneously (Supplementary Fig. 5). As a result, every colloidal particle has four nearest neighbors with the approximate arrangements with respect to $\mathbf{n}_0$ depicted in the Supplementary Fig. 5a. Arrangements that are mirror-symmetric with respect to $\mathbf{n}_0$ as well as the ones with inversion symmetry are also possible because of the non-polar uniaxial symmetry of the NLC host fluid. Upon formation of such a five-particle assembly, similar geometric constraints on attractive interactions of the peripheral particles within the assembly lead to additions of new particles at well-defined peripheral sites and eventually prompt formation of a regular two-dimensional lattice with a rhombic elementary cell (Supplementary Fig. 5b). Minimization of the cell's energy over the angles α and β defined in the Supplementary Fig. 5b yields their values, $\alpha \approx \theta_1$ and $\beta \approx \theta_2$, that correspond to the minima of the pairwise interaction determined in the main text. Hence, the ensuing colloidal crystal structure is expected to be driven mainly by interactions between the nearest neighbors. Extension of this analysis based on pair-interactions to three dimensions points into the possibility of interesting three-dimensional colloidal assemblies. For example, the planar five-particle assembly shown in Supplementary Fig. 5a can be readily transformed into a tetrahedron via rotation of the particles A and B around the z-axis by approximately 90°. Under such a transformation, θ_A and θ_B do not change and the energy of the colloidal unit cell remains close to its minimum. Hypothetically, the tetrahedrons can be subsequently assembled into a three-dimensional crystal lattice. Given the

angular dependence of U_{int} and the shape of two-dimensional elementary cell, one can also expect existence of three-dimensional colloidal crystals with low-symmetry (e.g. triclinic) lattices. Our highly over-simplified analysis here is of course based on pair interactions, but many-body and kinetic effects may become important in defining three-dimensional assemblies and will be of great interest to explore in future studies.

Supplementary Note 2 - Interaction potential of elastic hexadecapoles

It is well established that the far-field nematic director distortions induced by colloidal particles can be represented by means of elastic multipoles[1,2]. One can find unknown multipole moments either from long-range asymptotic of exact solutions for **n**(**r**) or from relevant experiments. Rigorously speaking, the multipole expansion given by Eq. (2) is an infinite series. In order to determine where it should be truncated, one can estimate the leading anharmonic correction F_{anh} to the free energy given by Eq. (1) associated with ∇n_z. Since the F_{har} is derived under the assumption of $n_z \approx 1$, one finds that $F_{anh} = \frac{K}{2}\int d\mathbf{r}(\nabla n_z)^2 \approx \frac{K}{8}\int d\mathbf{r}\left(\nabla n_\perp^2\right)^2$, where $n_\perp^2 = n_x^2 + n_y^2$. This modifies the Euler-Lagrange equations to become

$$\Delta n_\mu + \frac{1}{2} n_\mu \Delta n_\perp^2 = 0\,. \qquad (4)$$

It follows from Eq. (4) that if the leading term of the multipole expansion falls off as r^{-n} then the leading anharmonic correction behaves as r^{-3n}. Therefore, all terms that decay faster than r^{-3n} can be omitted within the scope of the harmonic approximation. Note also that at the same time all odd moments in Eq. (4) vanish as long as the director field is symmetric about the particle center[2-5]. Thus, a colloidal particle of quadrupole symmetry (Fig. 4a and b) is characterized by a set of three coefficients b_2, b_4 and b_6. In the case of beads with usual planar (Fig. 4a and d) or homeotropic (Fig. 4b and e) boundary conditions, the higher-order terms b_4 and b_6 are suppressed by the dominant quadrupolar one, b_2, so that their influence is substantial only at very short inter-particle distances. However, this

analysis does not hold for the particles with conically degenerate surface anchoring. The director in the vicinity of such a bead is a superposition of the two pure quadrupolar configurations separately containing the "Saturn ring" or boojums. For the cases of pure elastic quadrupoles, the former was characterized by[6] $b_2 = 0.4$ while the latter was found to have[2] $b_2 = -0.36$. Thus, the net quadrupole moment of our elastic multipole is small and can be tuned to zero by controlling the conical boundary conditions, so that the higher-order multipoles manifest themselves in a wider range of distances. To verify this scenario, we measured the time dependence of the inter-particle separation between two PSMs (Fig. 3a and Supplementary Fig. 6). The force of interaction between the particles, $\mathbf{F}_{int} = -\nabla U_{int}$, incorporates multipole coefficients b_2, b_4 and b_6 as adjustable parameters, which are found from the experimentally measured $R(t)$. Only a single, unique set of coefficients b_2, b_4 and b_6 provides satisfactory agreement between theoretical and experimental data curves (Fig. 3a and Supplementary Fig. 6), while also explaining the angular dependencies of interactions (Fig. 2).

The analysis of elastic interactions based on multipole expansion has its own limitations. A colloidal particle in the NLC host is surrounded by a small region where the director deviations are large and described by highly nonlinear equations[2]. Elastic interactions in the corresponding range of separations of $R < 2.4r_0$ are, therefore, also characterized by strong nonlinearities. In this region the multipole expansion is not valid and the present theoretical approach does not apply, so we exclude this region from our analysis of experimental data. From fitting experimental data (Fig. 3a and Supplementary Fig. 6), we find that the quadrupole moment $a_2 = b_2 r_0^3 = -0.017 r_0^3$ is indeed orders of magnitude smaller than that for quadrupolar NLC colloidal spheres with usual tangential or perpendicular surface anchoring. This yields an angular dependence of interactions that is essentially different from that of elastic dipoles and quadrupoles and consists of eight angular sectors of attraction separated by eight sectors of repulsion (Fig. 2a). The interaction energy has two minima in each quadrant (Fig. 2b), with angular positions of minima dependent on the separation R. For instance, when $R = 2.4r_0$ the corresponding minimum-energy angles are approximately 34° and 72° (Fig. 2b).

The slight difference between these values and the experimentally measured angles of assemblies $\theta_1 \approx 22°\text{-}26°$ and $\theta_2 \approx 64°\text{-}75°$ is caused by nonlinear distortions concentrated at the region between r_0 and about $1.2r_0$ from the center of every particle, causing complex nonlinear interactions at $R < 2.4r_0$ that are not captured within the theoretical framework described here.

Supplementary References

1. Lubensky, T. C., Pettey, D., Currier, N. & Stark, H. Topological defects and interactions in nematic emulsions. *Phys. Rev. E* **57**, 610–625 (1998).

2. Chernyshuk, S. B. High-order elastic terms, boojums and general paradigm of the elastic interaction between colloidal particles in the nematic liquid crystals. *Eur. Phys. J. E* **37**, 6 (2014).

3. Lev, B. I., Chernyshuk, S. B., Tomchuk, P. M. & Yokoyama, H. Symmetry breaking and interaction of colloidal particles in nematic liquid crystals. *Phys. Rev. E* **65**, 021709 (2002).

4. Tovkach, O. M., Chernyshuk, S. B. & Lev, B. I. Theory of elastic interaction between arbitrary colloidal particles in confined nematic liquid crystals. *Phys. Rev. E* **86**, 061703 (2012).

5. Chernyshuk, S. B., Tovkach, O. M. & Lev, B. I. Elastic octopoles and colloidal structures in nematic liquid crystals. *Phys. Rev. E* **89**, , 032505 (2014).

6. Ognysta, U. M., Nych, A. B., Uzunova, V. A., Pergamenschik, V. M., Nazarenko, V. G., Škarabot, M. & Muševič. Square colloidal lattices and pair interaction in a binary system of quadrupolar nematic colloids. *Phys. Rev. E* **83**, 041709 (2011).
